# Advanced oxidation processes for 2,4-dichlorophenoxyacetic acid in aqueous media: evidence gaps, mechanistic insights and emerging directions

**DOI:** 10.1098/rsos.260598

**Published:** 2026-10-14

**Authors:** Daniel H. Mendoza-Camarena, Yair Cruz-Narváez, José Javier Castro-Arellano, David Correa-Coyac, Raúl Suárez-Parra, Isaías Hernández-Pérez

**Affiliations:** ^1^ Department of Basic Sciences, Universidad Autónoma Metropolitana–Azcapotzalco, Azcapotzalco, Ciudad de México, Mexico; ^2^ Laboratorio de Posgrado e Investigación de Operaciones Unitarias, ESIQIE, Instituto Politécnico Nacional, UPALM, Zacatenco, Ciudad de México, Mexico; ^3^ Escuela Superior de Cómputo, Instituto Politécnico Nacional, Ciudad de México, Mexico; ^4^ Instituto de Energías Renovables, Universidad Nacional Autónoma de México, Temixco, Morelos, Mexico

**Keywords:** advanced oxidation processes, photocatalysis, 2,4-dichlorophenoxyacetic acid, water treatment, reactive oxygen species, electrochemical oxidation, persulfate activation, environmental remediation, 2,4-D, mineralization

## Abstract

The herbicide 2,4-dichlorophenoxyacetic acid (2,4-D) remains a relevant aquatic contaminant because parent-compound disappearance does not imply mineralization, detoxification or treatment readiness. This systematic review synthesizes recent evidence on advanced oxidation processes (AOPs) and closely related chemically coupled routes for aqueous 2,4-D treatment. Records from Scopus, Web of Science and Google Scholar within a documented 2020–2025 review window were screened using explicit eligibility criteria and assessed with an evidence-completeness rubric covering key reporting domains for cross-study comparison. The final corpus comprised 204 studies across photocatalytic/photo-assisted, electrochemical/electro-Fenton, Fenton-type, sulfate-radical, ozone-based, sonochemical, plasma-assisted and hybrid systems. Parent 2,4-D removal or analytical confirmation was reported in 202 studies, while carbon-based mineralization or organic-load evidence was reported in 116, transformation products in 95, residual toxicity in 27, real-water validation in 67 and reactor-scale or transferability information in 37. These patterns show stronger evidence for parent-compound disappearance than for treatment completeness, residual hazard and transferability. No AOP family was universally superior across removal, mineralization, toxicity reduction, matrix robustness, energy demand, durability and scale readiness. Future studies should standardize reporting of mineralization, transformation products, toxicity, energy boundaries, leaching, durability and reactor descriptors.

## 1. Introduction

The herbicide 2,4-dichlorophenoxyacetic acid (2,4-D) remains an environmentally relevant phenoxy herbicide because it is still detected in water-related matrices and exposure studies. Recent monitoring work has reported current-use pesticides, including 2,4-D, in waste water and urban or riverine waters, indicating that conventional collection and treatment systems may provide only partial abatement for this compound class [1,2]. Regional water-quality studies in agricultural areas further support the need to evaluate pesticide occurrence under conditions relevant to surface-water and drainage systems [3,4]. Human biomonitoring studies have also reported urinary 2,4-D in children and pregnant women, linking environmental occurrence with exposure assessment rather than treating the herbicide only as an analytical target [5–7]. Its environmental relevance, therefore, extends beyond the disappearance of the parent compound from a treated solution and includes transformation products, ecological effects and possible residual toxicity after treatment, as suggested by mesocosm and aquatic-toxicity studies involving 2,4-D-containing exposures [8–10].

A central difficulty in evaluating advanced oxidation process (AOP) studies is that parent-compound loss does not by itself demonstrate mineralization or detoxification. This distinction is especially important for 2,4-D because measurable disappearance can be misinterpreted as complete treatment when total organic carbon (TOC), chemical oxygen demand (COD), dechlorination, transformation products or toxicity endpoints are not reported. Comparative work on 2,4-D and 2,4-dichlorophenol in a planar falling-film reactor has shown that ozonation, photocatalysis and non-thermal plasma can differ markedly in parent-compound removal, mineralization and by-product persistence under reactor-comparable conditions [11]. Recent primary studies reinforce the same point across other AOP families: heterogeneous Fenton-like degradation over Fe${}^{0}$@Fe${}_{3}$O${}_{4}$ nanoparticles achieved complete 2,4-D removal while reaching only partial mineralization and detecting 2,4-dichlorophenol as a principal intermediate [12]; hybrid sonocatalytic and sonophotocatalytic treatments have evaluated mineralization and economic performance in addition to removal efficiency [13], and ultrasound-assisted electroactivation of persulfate (PS) has incorporated by-product analysis into the assessment of 2,4-D degradation [14]. In this review, *removal* refers to the loss of measurable parent 2,4-D, *degradation* refers to chemical transformation of the molecule and *mineralization* requires carbon-based evidence such as TOC or COD reduction.

This distinction between disappearance, transformation and mineralization also determines how AOPs should be compared. AOPs do not represent a single treatment mechanism; they comprise a family of activation routes in which chemical reagents, light, electric current, ultrasound, catalysts, plasma discharge or coupled configurations generate reactive species able to transform recalcitrant organic pollutants [15–17]. Depending on the system, 2,4-D oxidation may involve hydroxyl radicals, sulfate radicals, superoxide, singlet oxygen, surface-bound oxidants, high-valent metal species, direct electron transfer, reactive chlorine species in chloride-containing matrices or sequential reductive–oxidative steps. The same radical label, therefore, does not imply the same mechanism, location, lifetime or selectivity. Accordingly, the present review organizes the evidence by AOP family and activation function, including reagent-activated systems, Fenton and Fenton-like routes, ozonation, sulfate-radical processes, electrochemical oxidation, electro-Fenton, photochemical and photocatalytic systems, sonochemical treatment, plasma-assisted oxidation and hybrid treatment trains.

The recent 2,4-D literature illustrates why this broader comparison is necessary. The evidence base has diversified from conventional reagent-driven oxidation towards heterogeneous Fenton-like catalysts, sulfate-radical activation, sonocatalytic and sonophotocatalytic systems, electrochemical and membrane-assisted reactors, intensified ozonation, plasma-driven oxidant generation, recoverable catalysts and coupled reductive–oxidative sequences. These developments expand the treatment toolbox, but they also complicate direct ranking because each process family is governed by different activation modes, reactive-species profiles, matrix sensitivities and scale-relevant constraints. Comparative assessment must therefore consider initial 2,4-D concentration, aqueous matrix, reactor geometry, oxidant dose, catalyst or electrode loading, irradiation source, current density, transformation products, TOC/COD removal, toxicity endpoints, energy demand, durability, leaching and reporting of uncertainty, rather than only best-case endpoint removal percentages.

Reactor configuration is part of this comparison, not a secondary engineering detail. Small-batch systems are useful for mechanistic control, but scale-relevant AOP operation depends on photon delivery, gas–liquid transfer, electrode spacing, hydraulic residence time, catalyst recovery, fouling, energy boundaries and contact between reactive species and target compounds. Falling-film reactors are a relevant example because they can intensify gas–liquid interfacial area, improve photon or plasma contact and enable direct comparison of ozonation, photocatalysis, plasma-assisted treatment and related hybrid systems under a common reactor geometry [18,19]. In this review, falling-film, divided-cell, membrane-assisted, continuous-flow and related configurations are considered when they inform AOP operation, reactor transferability or scale-relevant comparison, without expanding the scope to membrane fabrication, electrospinning or packaging-material applications. Previous reviews provide an important basis for this field. Girón-Navarro *et al.* [20] reviewed AOPs for 2,4-D degradation and compared ozonation, photocatalytic, Fenton-type and electrochemical processes in terms of operating conditions, degradation efficiency, mineralization, intermediates and selected energy cost considerations. More recent pollutant-oriented reviews have broadened the discussion towards PS activations, nano zero-valent metal catalysts, degradation pathways, mineralization efficiency, catalyst recovery, secondary pollution, hybrid AOPs, low-cost activators and large-scale application challenges [17,21]. Taken together, these contributions show that AOP-based treatment can no longer be evaluated only through endpoint removal percentages. For 2,4-D in aqueous media, the recent literature still needs to be synthesized in a way that separates parent-compound disappearance from mineralization and detoxification, evaluates the completeness and availability of the supporting evidence domains and compares technology-relevant endpoints across process families, including real-water validation, catalyst or electrode durability, leaching, energy demand and reactor transferability.

The present review examines peer-reviewed records captured within the documented 2020–2025 review window that investigated AOP-based or closely coupled treatment routes for 2,4-D in aqueous media, with temporal-metadata exceptions handled transparently as described in the Methods and supplementary material. The scope is restricted to systems in which chemical transformation is central to performance, including oxidative, reductive–oxidative and coupled transformation processes. General water-contamination surveys, adsorption-only phase transfer and purely biological remediation were not included in the systematic evidence corpus. Adsorption, membrane separation or catalytic materials with sorptive capacity were considered only when the study experimentally evaluated an oxidative or coupled chemical transformation contribution. This delimitation preserves a focused comparison of treatment systems while still allowing reactor-relevant configurations and hybrid processes to be discussed where they directly affect 2,4-D degradation, mineralization, toxicity reduction or technology transfer.

The objective of this review is twofold. First, it summarizes recent process-level advances in AOP-based degradation of 2,4-D across the main treatment families reported in the 2020–2025 literature. Second, it identifies the methodological and technological gaps that still limit comparison, evidence grading and translation beyond controlled laboratory conditions. By combining a structured search strategy, explicit eligibility criteria, evidence-completeness assessment and semi-quantitative synthesis, this review aims to clarify which AOP families appear promising for specific endpoints, how robust the supporting evidence is and which information remains necessary for environmentally meaningful deployment.

## 2. Methods

### 2.1. Review question and scope

This systematic review was designed to synthesize the evidence on AOPs and closely related chemically coupled treatment routes for 2,4-D in aqueous media. The operational review question was: *How effective are AOP-based and coupled treatment systems at degrading, mineralizing or reducing the residual toxicity of 2,4-D in aqueous matrices, and which operational, mechanistic and scale-relevant factors determine their performance?*

The question was structured using a framework inspired by population, intervention, comparator, outcomes and study design (PICOS) and population, exposure, comparator and outcome (PECO), adapted to laboratory and bench-scale studies in environmental chemistry. This approach followed the use of structured eligibility components in systematic reviews and the PECO framework for environmental exposure questions [22,23]. In this adaptation, the population or system was defined as aqueous matrices containing 2,4-D. These matrices included model water, synthetic matrices, surface water, groundwater, waste water, agricultural drainage and other complex waters.

The intervention component comprised AOP-based or closely coupled treatment routes. These included Fenton and Fenton-like systems, photo-Fenton, electro-Fenton, anodic oxidation, ozonation, semiconductor photocatalysis, PS or peroxymonosulfate (PMS) activation, sonochemical treatment, plasma-assisted processes, photoelectrochemical systems and hybrid treatment trains. Catalytic reductive, reductive–oxidative and emerging coupled routes were retained only when they targeted chemical transformation of 2,4-D in water and reported outcomes comparable with the AOP dataset.

Comparators, when available, included untreated controls, oxidant-only systems, catalyst-only systems, photolysis-only systems, alternative AOP families, model versus real-water matrices and the presence or absence of scavengers, natural organic matter or inorganic ions. The primary outcome domains were parent 2,4-D removal or degradation, mineralization evidenced by TOC or COD and detoxification or residual toxicity when reported. Additional mechanistic and technology-relevant outcomes included dechlorination, kinetic descriptors, transformation products, reactive-species evidence, catalyst or electrode reuse, leaching, energy demand, current efficiency, real-water validation and reactor configuration.

Throughout this review, parent-compound disappearance is distinguished from mineralization. The former refers to the loss of measurable 2,4-D, whereas the latter requires TOC/COD reduction or equivalent carbon-based evidence. This distinction is necessary because chlorinated intermediates or low-molecular-weight oxidation products can persist after the parent compound is no longer detected; therefore, removal of measurable 2,4-D is not treated here as evidence of complete treatment unless supported by mineralization or toxicity endpoints [11,12].

### 2.2. Information sources and search strategy

The review followed a PRISMA 2020-guided structure for study identification, screening and reporting [22]. The predefined review window for eligibility was 2020–2025. Searches were conducted in Scopus, Web of Science Core Collection and Google Scholar, and the final retrieval workflow, documentation and corpus reconciliation were completed on 13 May 2026. This documentation date describes workflow completion, whereas the eligibility window remained 2020–2025.

Corpus definition followed the documented retrieval workflow and eligibility criteria. Records showing 2026 issue-year metadata were retained only when they had been captured by the documented search, met all eligibility criteria and corresponded to online-first publication or bibliographic metadata updates. These temporal-metadata exceptions are listed in the supplementary material.

The search strategy was designed to retrieve studies on AOP-based or chemically coupled transformation of 2,4-D in aqueous media. It combined terms for the target compound, AOP families, treatment outcomes and aqueous matrices. The AOP family block included photocatalysis, Fenton and photo-Fenton processes, electro-Fenton, electrochemical oxidation, anodic oxidation, ozonation, PS and PMS activation, sulfate-radical processes, sonochemistry, plasma-assisted treatment and falling-film configurations. The outcome block included degradation, mineralization, TOC, COD, toxicity, intermediates and kinetics. Search strings were adapted to the syntax of each database, including field tags, year limits, document-type filters and language filters.

The core search string was:

(“2,4-D” OR “2,4-dichlorophenoxyacetic acid” OR “dichlorophenoxyacetic acid”) AND (“advanced oxidation” OR AOP OR photocatal* OR “photo-Fenton” OR Fenton OR “electro-Fenton” OR electrooxidation OR “electrochemical oxidation” OR “anodic oxidation” OR ozonation OR ozone OR persulfate OR peroxymonosulfate OR PMS OR PS OR “sulfate radical” OR sonochem* OR ultrasound OR plasma OR “falling film”) AND (degrad* OR mineraliz* OR TOC OR COD OR toxicity OR intermediates OR kinetics) AND (water OR aqueous OR wastewater OR groundwater OR “real water”)

Studies centred only on adsorption or phase transfer were excluded during screening and eligibility assessment. Coupled adsorption–oxidation, catalyst-assisted or sorptive-material systems were retained when the study experimentally evaluated oxidative, reductive–oxidative or otherwise coupled chemical transformation of 2,4-D in water.

In Scopus, the query was implemented using TITLE-ABS-KEY fields and restricted to publication years 2020–2025, document type *Article*, language *English* and source type *Journal*, yielding 140 records. In Web of Science Core Collection, the equivalent query was implemented using TS fields with the same year, document-type and language restrictions, yielding 204 records.

Google Scholar was searched manually using shorter process-specific query variants, sorted by relevance within the custom range 2020–2025. For each query variant, up to the first 100 results or 10 pages were screened, unless two consecutive pages produced no potentially eligible records. These Google Scholar searches yielded 256 records captured for cross-source deduplication and title/abstract screening.

Table 1 summarizes the database-specific retrieval parameters included in the review. Complete search strings, Google Scholar query variants, screening depth, stopping points and export details are provided in supplementary material 1.

**Table 1.** Database-specific retrieval parameters used to construct the systematic review corpus. Complete search strings, Google Scholar query variants, screening depth, stopping points and export details are provided in supplementary material 1.

source	retrieval parameters	Records
Scopus	TITLE-ABS-KEY Boolean query adapted to the target compound, AOP families, outcomes and aqueous matrices; restricted to 2020–2025, *Article*, English and journal source type; retrieval workflow documented on 13 May 2026	140
Web of Science Core Collection	TS Boolean query equivalent to the Scopus search blocks and adapted to database syntax; restricted to 2020–2025, article-type records and English-language records; retrieval workflow documented on 13 May 2026	204
Google Scholar	manual process-specific query variants because equivalent field-controlled Boolean searching was not available; custom range 2020–2025; relevance sorting; up to 100 results or 10 pages per variant, with the stopping rule documented in supplementary material 1	256

### 2.3. Record management and deduplication

Records retrieved from Scopus, Web of Science and Google Scholar were imported into Zotero and organized in source-specific collections. Raw source collections and search exports were preserved as part of the auditable review record. The merged search set contained 600 records before deduplication: 140 from Scopus, 204 from Web of Science and 256 from Google Scholar.

Deduplication was performed in Zotero and verified by reconciliation with the Rayyan screening export. Potential duplicates were checked using DOI, title, author list, publication year, journal metadata and, when necessary, manual comparison of duplicate-version records. This process identified 285 duplicate or duplicate-version records. After deduplication, 315 unique records remained for title and abstract screening. The deduplication and reconciliation records are provided in the supplementary material.

### 2.4. Eligibility criteria

Studies were eligible when they were peer-reviewed primary research articles in English, captured within the documented 2020–2025 review window, investigated 2,4-D as an explicit target compound or an individually quantified target compound and evaluated chemical transformation in an aqueous matrix or water-treatment configuration. Eligible interventions included AOPs and closely related chemically coupled routes, including Fenton and Fenton-like processes, photochemical and photocatalytic oxidation, electrochemical oxidation, electro-Fenton, sulfate-radical processes, ozonation, sonochemical treatment, plasma-assisted oxidation, catalytic redox routes and hybrid treatment trains. To be retained, studies had to report at least one quantitative treatment outcome linked to 2,4-D transformation, such as parent-compound removal or degradation, mineralization, dechlorination, kinetic behaviour, transformation products or residual toxicity. Technology-relevant domains, including reuse, leaching, stability, energy consumption, current efficiency and reactor performance, were extracted when available but were not sufficient as stand-alone eligibility criteria in the absence of a 2,4-D treatment outcome.

Studies were excluded when they were reviews, book chapters, editorials, conference abstracts without full peer-reviewed data, non-aqueous studies, purely biological treatments, adsorption-only or phase-transfer-only systems or studies in which 2,4-D was not an explicit and extractable target compound. Adsorption was considered within scope only when it was part of a coupled oxidative or redox treatment system, and the chemical transformation of 2,4-D was experimentally evaluated. Membrane fabrication, electrospun packaging materials and membrane-morphology studies were excluded unless the membrane or separator was directly involved in AOP operation, divided-cell operation, mass transfer, catalyst/electrode separation or scale-relevant water-treatment performance.

### 2.5. Screening and study selection

Title and abstract screening was conducted in Rayyan by two reviewers using predefined eligibility categories. Records were classified as eligible, potentially eligible or excluded at the title/abstract stage. The potentially eligible category was used when the title and abstract did not provide enough information to confirm the target compound, process type, aqueous matrix, publication type or outcome domain. Discrepant or uncertain records were resolved by discussion until consensus was reached. Reviewer-level blinded decisions were not archived before consensus in a form suitable for reliable retrospective calculation of Cohen’s kappa or percentage agreement. The process is therefore reported as a two-reviewer consensus-based screening workflow. To reduce the risk of false exclusion, all records classified as eligible or potentially eligible were advanced to full-text retrieval.

The title/abstract screening set contained 315 unique records, with no undecided records remaining after screening. Of these, 210 were judged eligible for retrieval, 10 were judged potentially eligible and 95 were excluded at title/abstract screening. Therefore, 220 reports were sought for retrieval. Three reports could not be obtained after full-text retrieval attempts and were recorded as not retrieved. The remaining 217 reports were assessed for full-text eligibility; 204 met the inclusion criteria, and 13 were excluded at full text. The final systematic evidence corpus therefore comprised 204 included studies. The study selection workflow and record counts are shown in figure 1.

**Figure 1.**
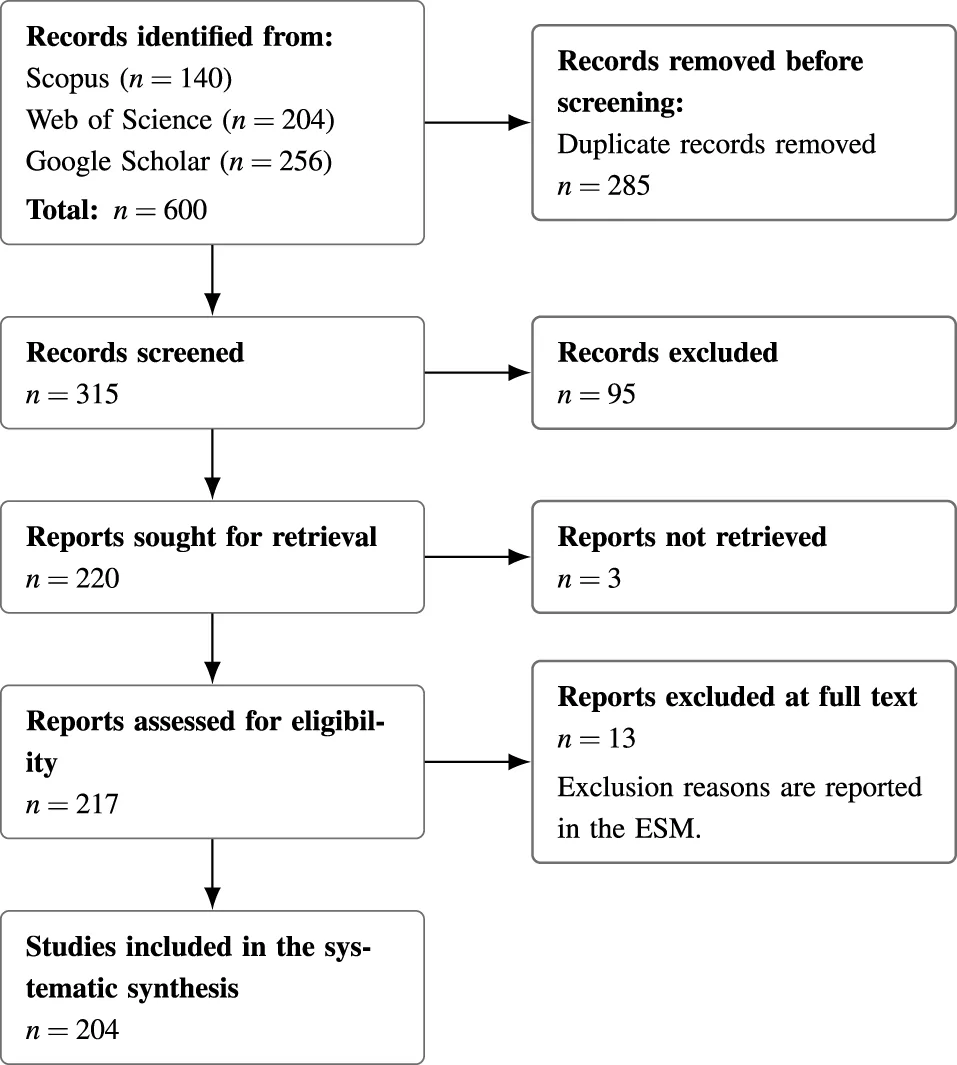
PRISMA 2020 flow diagram for study selection in the systematic review of AOP-based and chemically coupled treatment routes for 2,4-D in aqueous media.

Screening decisions and operational labels were exported from Rayyan and reconciled with Zotero using record keys and bibliographic metadata. The Rayyan export, title/abstract decision counts and Zotero collection structure were preserved as part of the review audit trail. Full-text eligibility decisions were recorded in the standardized extraction workbook, including inclusion or exclusion decision, exclusion rationale where applicable, AOP family coding and reporting-domain classification. The full screening, retrieval and eligibility records are provided in the supplementary material.

### 2.6. Data extraction

Data were extracted from each of the 204 included studies using a standardized full-text assessment and synthesis workbook. Extracted variables covered bibliographic information, AOP family and subtype, reactor configuration, aqueous matrix, initial 2,4-D concentration, pH, oxidant dose, catalyst or electrode composition and loading, light source or electrical input, electrolyte where applicable, treatment time, analytical method, parent 2,4-D removal, TOC or COD mineralization, dechlorination, kinetics, transformation products, residual toxicity, reuse, leaching, post-use catalyst/electrode/material characterization, energy or current-efficiency metrics and scale-relevant reactor descriptors such as reactor volume, flow rate or residence time. Variables were normalized where possible, while original reported units and study-level details were retained in the supplementary material.

AOP families were coded using two criteria: the dominant activation route and the technological function. The resulting groups were Fenton and Fenton-like systems; photo-Fenton and photoelectro-Fenton systems; electrochemical and electro-Fenton systems; ozone-based and catalytic ozonation systems; sulfate-radical systems based on PMS or PS activation; photochemical, photocatalytic and other photo-assisted systems; sonochemical and sonophotochemical systems; plasma-assisted systems and coupled reductive–oxidative or hybrid treatment systems. Emerging and hybrid routes were assigned according to their functional role, including radical generation, reductive–oxidative sequencing, mass-transfer intensification, autonomous or reactor-assisted motion, plasma-driven oxidant formation and passive release of reactive species. This functional coding distinguished mechanistically different emerging and hybrid routes rather than treating them as a residual category.

Outcome availability was coded by the evidence domain to support cross-study comparison. Parent-compound removal, mineralization, transformation products, residual toxicity, kinetics, matrix complexity, energy or cost metrics, stability/reuse/leaching, reactor transferability and reporting transparency were coded explicitly to distinguish high removal efficiency from broader evidence of treatment completeness and practical transferability.

The complete bibliography of the 204 included studies is provided as a dedicated supplementary material file and linked to the row-level extraction, eligibility and evidence-assessment records. In the main text, representative studies are cited when discussing family-level or endpoint-specific patterns.

### 2.7. Evidence-completeness and reporting-domain assessment

A domain-specific evidence-completeness framework was used to support the synthesis of heterogeneous laboratory and bench-scale AOP studies. The framework was designed to evaluate the availability and completeness of evidence domains relevant to treatment assessment and cross-study comparison. The framework is summarized in table 2, and the complete study-by-study scoring table is provided in supplementary material 4.

**Table 2.** Domain-specific evidence-completeness framework used to support systematic synthesis and cross-study comparison. The complete study-by-study scoring table is provided in supplementary material 4.

criterion	0	1	2
analytical confirmation	UV–visible alone or another non-selective signal	partial chromatographic confirmation or endpoint selective analysis	selective quantification by HPLC, GC, LC–MS or HRMS with appropriate controls
mineralization	no TOC/COD or equivalent carbon-based evidence	endpoint TOC/COD only	temporal TOC/COD data and explicit mineralization discussion
transformation products	not reported	proposed pathway with limited intermediate evidence	MS/HRMS-supported intermediates with cautious pathway interpretation
residual toxicity	not assessed	single bioassay or limited endpoint	bioassay with controls and before/after comparison
kinetics	endpoint removal percentage only	basic kinetic model	kinetic model with replicates, error or robust comparative analysis
matrix complexity	ultra-pure or model water only	synthetic matrix with scavengers or selected ions	real water or validated complex matrix
energy or cost metrics	not reported	partial metric	kWh m${}^{-3}$, electrical energy per order, current efficiency, life-cycle indicator or cost estimate
stability, reuse and leaching	no reuse or stability test	reuse tested without leaching assessment	reuse plus leaching assessment or post-use characterization
reactor scale or transferability	small-batch test only	laboratory flow, divided-cell, membrane-assisted or continuous configuration	scale-relevant reactor, falling-film or pilot-scale configuration with transferable operating descriptors
reporting transparency	no replicates, error or accessible support data	partial replicates or incomplete uncertainty reporting	replicates, error bars, accessible supporting data or sufficient methodological detail

Each study was scored from 0 to 2 across 10 domains: analytical confirmation of parent 2,4-D, mineralization evidence, transformation-product identification, residual toxicity, kinetic treatment, matrix complexity, energy or cost reporting, stability, reuse and leaching, reactor scale or transferability and reporting transparency. Total scores were used to classify the evidence available for synthesis as limited evidence completeness (0–9), moderate evidence completeness (10–15) or high evidence completeness (16–20). These categories were used to guide the strength and scope of synthesis statements across the corpus.

The framework evaluates reporting completeness and evidence-domain availability. It was not designed or interpreted as a formal risk-of-bias instrument, a direct measure of internal validity or an overall ranking of methodological robustness. Because the score reflects the number and depth of reported evidence domains, studies reporting a broader endpoint set may obtain higher evidence-completeness scores even when their experimental design is not necessarily more robust.

### 2.8. Synthesis strategy and rationale for not conducting meta-analysis

A formal pooled-effect meta-analysis was not statistically defensible because the included studies were highly heterogeneous in initial 2,4-D concentration, aqueous matrix, reactor configuration, catalyst or electrode material, oxidant dose, irradiation or current regime, analytical endpoint, treatment time and uncertainty reporting. This decision follows methodological guidance that pooled meta-analysis should be avoided when studies are not sufficiently similar to support a meaningful common effect estimate, and that structured synthesis without meta-analysis should transparently report grouping, heterogeneity and evidence-strength criteria [24,25].

Studies were grouped by AOP family and by mechanistic or technological function. Cross-cutting comparisons were made across parent-compound removal, TOC/COD mineralization, dechlorination, transformation products, residual toxicity, matrix effects, energy demand, durability, leaching, real-water validation and scale-relevant reactor configurations. The interpretation of each pattern was guided by the evidence-completeness classification. Consequently, conclusions were framed according to the strength and comparability of the available evidence, without presenting them as pooled estimates of treatment performance.

### 2.9. Reproducibility and supplementary materials

The supplementary material and the associated Open Science Framework project provide the materials needed to reconstruct the systematic review workflow, including the database-specific search strategies, Google Scholar search log, Zotero and Rayyan screening records, deduplication and reconciliation records, PRISMA counts, full-text retrieval and exclusion records, data-extraction template, evidence-completeness assessment table and complete bibliography of included studies. These materials document how records were identified, deduplicated, screened, retrieved, assessed, extracted, scored and synthesized.

## 3. Results

### 3.1. Corpus overview and evidence-completeness profile

The final systematic synthesis comprised 204 included studies. These studies constitute the denominator for all corpus-level percentages reported below. Full-text exclusions and reports not retrieved are documented in the PRISMA workflow and in the supplementary material retrieval and full-text eligibility logs.

The included corpus was unevenly distributed among AOP families (table 3). Photocatalytic and photo-assisted systems represented the largest subset, with 105 of 204 studies (51.5%). Sulfate-radical systems based on PMS or PS activation accounted for 29 studies (14.2%), followed by electrochemical and electro-Fenton systems with 27 studies (13.2%) and ozone-based or catalytic ozonation systems with 16 studies (7.8%). Fenton/Fenton-like, photo-Fenton/photoelectro-Fenton, sonochemical/sonophotochemical and plasma-assisted systems were represented by smaller subsets. One study was coded as a coupled reductive–oxidative or hybrid-train configuration.

**Table 3.** Final distribution of included studies by normalized AOP family and evidence-completeness category. Percentages are calculated using the final included corpus as denominator (n=204). Evidence-completeness categories were assigned from the 0–20 domain-specific score: limited evidence completeness, 0–9; moderate evidence completeness, 10–15; high evidence completeness, 16–20. EC, evidence completeness.

AOP family	studies	share (%)	limited EC	moderate EC	high EC	mean score
photocatalysis/photo-assisted	105	51.5	77	28	0	7.8
sulfate-radical/PMS–PS	29	14.2	10	19	0	10.7
electrochemical/electro-Fenton	27	13.2	7	19	1	11.4
ozone-based/catalytic ozonation	16	7.8	8	8	0	9.8
Fenton/Fenton-like	10	4.9	6	4	0	9.1
photo-Fenton/photoelectro-Fenton	6	2.9	4	2	0	8.3
sonochemical/sonophotochemical	5	2.5	2	3	0	10.6
plasma-assisted	5	2.5	2	3	0	10.2
coupled reductive–oxidative/hybrid train	1	0.5	1	0	0	9.0
total	204	100.0	117	86	1	9.1

This distribution shows that the recent 2,4-D AOP literature is numerically concentrated in light-driven heterogeneous and photo-assisted systems. Consequently, family-level comparisons are most informative for photocatalytic/photo-assisted, sulfate-radical and electrochemical/electro-Fenton systems. Percentages for smaller families should be interpreted cautiously because they are sensitive to small sample size.

The evidence-completeness profile was also uneven. Of the 204 included studies, 117 fell in the limited evidence-completeness category, 86 in the moderate category and one in the high category. These categories reflect the extent to which studies reported the domains required for cross-study interpretation, including selective analytical confirmation, mineralization, transformation products, residual toxicity, matrix validation, energy demand, durability, leaching and reactor transferability information. They should therefore be interpreted as levels of evidence completeness for systematic synthesis, not as absolute judgements of individual study quality.

Among the larger families, electrochemical/electro-Fenton systems showed the most complete mean reporting profile, followed by sulfate-radical systems. Photocatalytic/photo-assisted studies formed the largest subset but had a lower mean evidence-completeness score, reflecting the frequent absence of toxicity, matrix validation and reactor-scale or transferability endpoints in this family. Smaller subsets, such as sonochemical/sonophotochemical and plasma-assisted systems, showed mean scores above the corpus average, but their sample sizes were too small to support broad family-level generalizations.

Taken together, the family distribution and evidence-completeness scores show that the corpus is large enough to identify cross-family reporting patterns, but not balanced enough to support simple technology rankings. The synthesis, therefore, treats AOP families as process families with distinct reporting profiles, strengths and limitations, rather than as directly interchangeable treatment options.

### 3.2. Outcome availability and reporting depth

Outcome reporting was highly uneven across the final corpus (table 4). Parent 2,4-D removal or analytical confirmation was reported in 202 of 204 studies (99.0%), making it the most consistently available endpoint. By contrast, TOC/COD evidence of mineralization or organic-load reduction was reported in 116 studies (56.9%). This gap shows that parent-compound disappearance was documented far more consistently than carbon- or oxygen-demand-based evidence of treatment completeness.

**Table 4.** Outcome availability across the final corpus of 204 included studies. Availability is defined as a non-zero score in the corresponding evidence-assessment domain. TOC/COD evidence includes reported mineralization or organic-load reduction, as applicable to the original study.

outcome or evidence domain	studies	share (%)
parent removal/analytical confirmation	202	99.0
TOC/COD evidence	116	56.9
intermediates/transformation products	95	46.6
residual toxicity	27	13.2
kinetics	180	88.2
matrix complexity/real-water validation	67	32.8
energy/cost	112	54.9
reuse/leaching/durability	139	68.1
reactor scale/flow/transferability	37	18.1

This imbalance is important because the outcome domains do not carry the same interpretive weight. Parent-compound removal establishes that measurable 2,4-D decreased under the tested conditions, but it does not show whether the organic carbon load was reduced, whether chlorinated intermediates persisted or whether the treated solution became less hazardous. TOC and COD data, therefore, provide a broader indication of treatment completeness than parent-compound removal alone, although COD-based evidence should still be interpreted as organic-load reduction rather than direct proof of full mineralization. For this reason, the availability of carbon-based endpoints was treated as a separate evidence domain in the synthesis.

Transformation products were reported in 95 studies (46.6%), while residual toxicity was assessed in 27 studies (13.2%). Residual toxicity therefore represented the weakest evidence domain in the corpus. Kinetic information was common, appearing in 180 studies (88.2%), but its reporting depth varied from basic endpoint fitting to analyses supported by uncertainty, replicates or comparative modelling. Matrix complexity or real-water validation was reported in 67 studies (32.8%), indicating that most systems were still evaluated in model waters or simplified matrices.

Technology-relevant outcomes were also incompletely reported. Energy or cost metrics were available in 112 studies (54.9%), reuse, leaching or durability in 139 studies (68.1%), and reactor scale, flow operation or transferability in 37 studies (18.1%). However, the reuse, leaching and durability domain should not be read as evidence that all three aspects were routinely assessed, because many studies reported reuse tests alone. Similarly, the presence of an energy or cost metric did not always imply comparable system boundaries across studies. These values show that practical comparison remains limited not only by treatment performance but also by inconsistent reporting of system boundaries, durability, leaching and reactor descriptors.

Taken together, these reporting patterns define the main interpretive constraint of the synthesis. The literature is extensive for demonstrating parent-compound disappearance, but less complete for evaluating mineralization, detoxification, real-water robustness and scale-relevant operation. The subsequent cross-family comparison therefore treats outcome availability as an evidence map rather than as a direct ranking of treatment performance.

### 3.3. Cross-family outcome patterns

Outcome-reporting profiles differed substantially across AOP families (figure 2; table 5). The heat map should be interpreted as a map of reporting completeness, not as a direct ranking of treatment performance. Darker cells indicate that a larger share of studies within a given family reported the corresponding evidence domain, whereas lighter cells identify endpoints that were less consistently documented. This distinction is important because a family may show frequent parent-compound removal while still providing limited evidence on mineralization, toxicity reduction, matrix robustness or reactor transferability.

**Figure 2.**
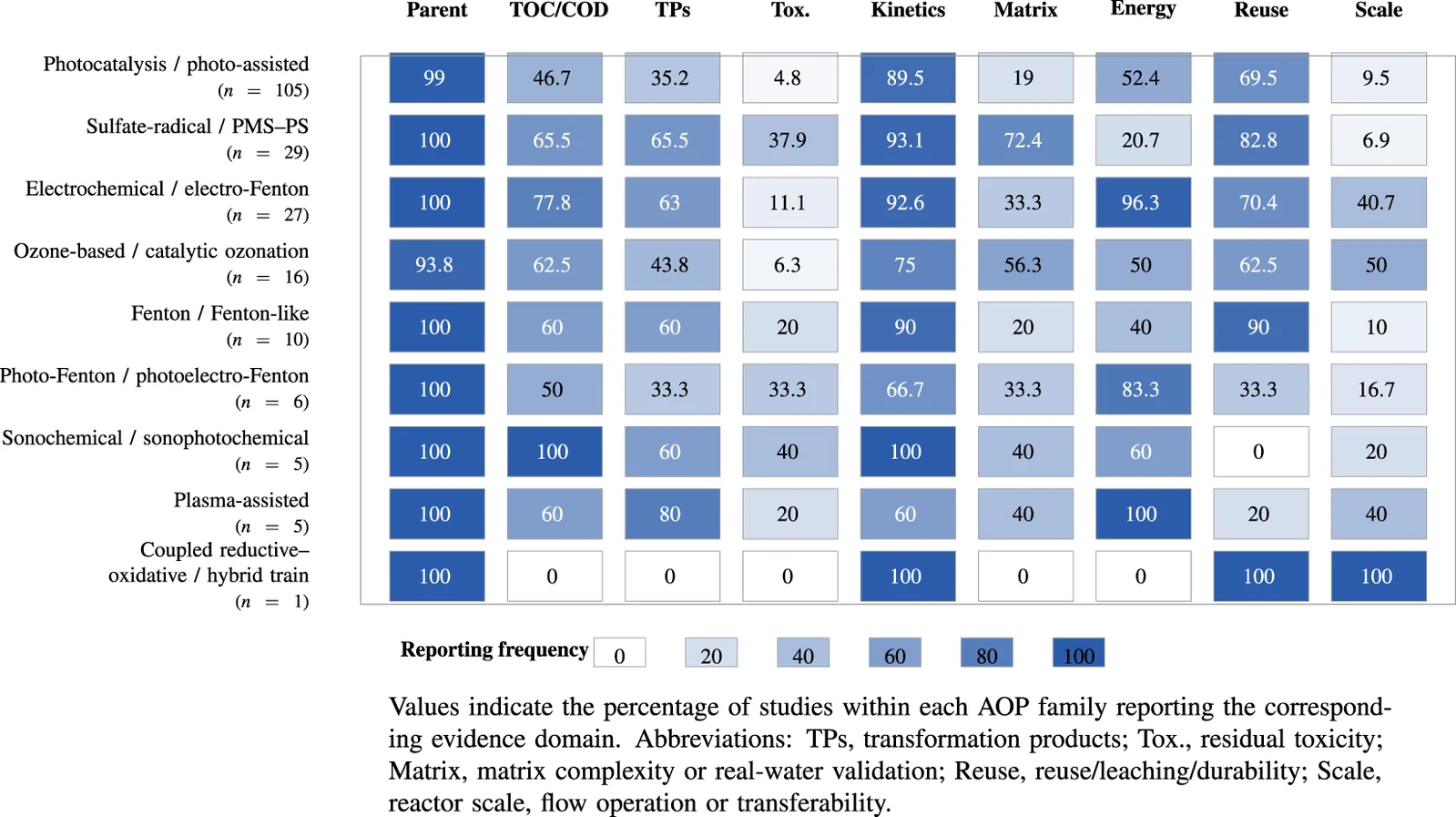
Heat map of outcome-reporting frequency across normalized AOP families in the final included corpus. Cell values represent the percentage of studies within each family reporting the corresponding evidence domain. The TOC/COD column denotes reported evidence of mineralization or organic-load reduction, as applicable to the original study. Larger families permit more robust cross-family interpretation, whereas smaller families should be interpreted cautiously because percentages are highly sensitive to sample size.

**Table 5.** Outcome-reporting percentages by AOP family in the final included corpus. Values are percentages of included studies within each family with a non-zero score in the corresponding evidence-assessment domain. Small families should be interpreted cautiously because percentages are sensitive to sample size. The underlying family-level counts are provided in the supplementary synthesis tables in the supplementary material.

AOP family	n	parent	TOC/COD	TPs	tox.	kin.	matrix	energy	reuse	scale
photocatalysis/photo-assisted	105	99.0	46.7	35.2	4.8	89.5	19.0	52.4	69.5	9.5
sulfate-radical/PMS–PS	29	100.0	65.5	65.5	37.9	93.1	72.4	20.7	82.8	6.9
electrochemical/electro-Fenton	27	100.0	77.8	63.0	11.1	92.6	33.3	96.3	70.4	40.7
ozone-based/catalytic ozonation	16	93.8	62.5	43.8	6.3	75.0	56.3	50.0	62.5	50.0
Fenton/Fenton-like	10	100.0	60.0	60.0	20.0	90.0	20.0	40.0	90.0	10.0
photo-Fenton/photoelectro-Fenton	6	100.0	50.0	33.3	33.3	66.7	33.3	83.3	33.3	16.7
sonochemical/sonophotochemical	5	100.0	100.0	60.0	40.0	100.0	40.0	60.0	0.0	20.0
plasma-assisted	5	100.0	60.0	80.0	20.0	60.0	40.0	100.0	20.0	40.0
coupled reductive–oxidative/hybrid train	1	100.0	0.0	0.0	0.0	100.0	0.0	0.0	100.0	100.0

The largest subset, photocatalysis and photo-assisted treatment, reported parent removal and kinetics frequently. However, this family showed weaker coverage of residual toxicity, matrix validation and reactor transferability. This pattern suggests that the most populated family in the corpus was also strongly shaped by proof-of-concept material studies and light-driven performance optimization. As a result, photocatalytic and photo-assisted systems provide extensive evidence for parent-compound disappearance, but less consistent evidence for environmental completeness and technology transfer.

Sulfate-radical systems based on PMS or PS activation showed stronger reporting of TOC/COD evidence, transformation products and matrix validation than most other larger families. This broader reporting profile is useful for interpreting mineralization, by-product formation and matrix sensitivity. However, energy and reactor-scale or transferability metrics were less consistently reported in this family, which limits direct comparison with more engineering-oriented AOP configurations.

Electrochemical and electro-Fenton systems showed a contrasting profile. Energy or cost metrics were reported in nearly all studies, and reactor-scale or transferability information was more common than in other larger families. This reflects the central role of current density, electrical input, electrode configuration and reactor operation in electrochemical AOPs. Nevertheless, residual toxicity and real-water validation remained limited, indicating that stronger energy or reactor reporting does not automatically imply a complete environmental assessment.

Ozone-based and catalytic ozonation studies showed a more evenly distributed reporting profile across TOC/COD, matrix, energy and scale domains, although toxicity evidence remained sparse. Smaller families, including Fenton and Fenton-like systems, photo-Fenton and photoelectro-Fenton systems, sonochemical and sonophotochemical systems and plasma-assisted systems, showed comparatively high percentages in some domains. These values should be interpreted cautiously because small sample sizes make the percentages highly sensitive to individual studies. The single coupled reductive–oxidative or hybrid-train study was retained because it met the eligibility criteria, but its percentages should not be interpreted as a family-level trend.

Overall, the cross-family comparison reinforces the central finding of the review: the recent literature does not support a universal hierarchy of AOP families. Instead, each family is better supported for some evidence domains than for others. Consequently, process comparison should be endpoint-specific: parent-compound removal, mineralization, toxicity reduction, matrix robustness, energy demand, durability and reactor transferability must be evaluated as separate but complementary dimensions.

### 3.4. Cross-cutting evidence gaps

The outcome- and family-level comparisons converged on six cross-cutting evidence gaps (table 6). These gaps concern the difference between parent-compound disappearance and mineralization, the scarce assessment of residual toxicity, uneven transformation-product and mechanistic support, limited real-water validation, incomplete reporting of energy, durability and scale descriptors and the predominance of limited-to-moderate evidence completeness across the corpus.

**Table 6.** Main cross-cutting evidence gaps identified from the final 204-study corpus and their implications for synthesis.

gap	evidence signal	interpretation for the synthesis
parent disappearance versus mineralization	parent removal or analytical confirmation: 202/204 studies (99.0%); TOC/COD evidence of mineralization or organic-load reduction: 116/204 studies (56.9%)	2,4-D disappearance should not be equated with complete treatment. Mineralization claims require carbon-based evidence; COD-based evidence indicates organic-load reduction
residual toxicity	residual toxicity: 27/204 studies (13.2%)	detoxification remains weakly supported relative to parent removal. Environmental-hazard claims require bioassay or comparable toxicity evidence
transformation products and mechanistic support	intermediates or transformation products: 95/204 studies (46.6%)	mechanistic pathways and ROS attribution should be framed according to the supporting evidence, not inferred from removal alone
matrix complexity and real-water validation	matrix or real-water validation: 67/204 studies (32.8%)	technology relevance remains limited when systems are optimized only in ultra-pure or simplified model waters
energy, durability and scale	energy/cost metrics: 112/204 studies (54.9%); reuse/leaching/durability: 139/204 studies (68.1%); reactor transferability: 37/204 studies (18.1%). The reuse/leaching/durability domain often reflected reuse testing alone	practical comparison requires compatible system boundaries, durability evidence, leaching assessment and reactor configurations beyond small-batch proof-of-concept tests
evidence completeness	final corpus: 117 limited-, 86 moderate- and 1 high-evidence-completeness studies	conclusions should be graded by evidence completeness and comparability, not by unqualified best-case performance claims

Together, these gaps define the boundary conditions for interpreting the review. The evidence base is extensive for demonstrating 2,4-D removal under optimized conditions, but less complete for determining whether treatment achieves mineralization, detoxification, matrix robustness, durability and transferability beyond small-batch operation.

## 4. Discussion

### 4.1. Principal findings and evidence-completeness profile

This systematic synthesis shows that the recent AOP literature on 2,4-D is large enough to identify robust reporting patterns, but not yet complete enough to support unqualified technology rankings. The final corpus of 204 studies was numerically dominated by photocatalytic and photo-assisted systems (table 3), indicating sustained interest in light-driven materials, catalyst design and tunable activation routes [26–28]. However, this dominance also suggests that the apparent direction of the field is strongly shaped by proof-of-concept material studies, whereas reactor-transferable, continuous-flow, plasma-assisted, sonochemical and explicitly coupled treatment configurations remain comparatively under-represented (figure 2; table 5) [13,29–32].

The evidence-completeness assessment changes the interpretation of this literature. Although most studies reported parent 2,4-D removal or degradation, the corpus was dominated by limited-to-moderate evidence-completeness profiles (table 3). This pattern shows that many studies do not report the combined information needed for cross-study environmental and technological assessment: selective analytical confirmation, TOC/COD evidence of mineralization or organic-load reduction, transformation products, residual toxicity, matrix validation, energy demand, durability, leaching and reactor-transferability descriptors.

The principal finding is therefore not that one AOP family is universally superior, but that the strength of the evidence depends strongly on the endpoint being considered. The field is comparatively mature in demonstrating parent-compound disappearance under optimized laboratory conditions. It is less mature in showing that this disappearance is accompanied by mineralization, detoxification, transformation-product control, matrix robustness, operational durability and scale-relevant performance (table 6). Consequently, the most defensible interpretation is evidence-graded: AOPs remain a promising toolbox for 2,4-D treatment, but claims of process superiority should be restricted to the domains actually supported by each study or process family.

### 4.2. Why parent-compound removal is not sufficient

The most important interpretive distinction in this review is between parent-compound removal, mineralization and detoxification. Parent 2,4-D removal or analytical confirmation was reported in 202 of 204 studies, whereas TOC/COD evidence of mineralization or organic-load reduction was reported in 116 studies and residual toxicity in only 27 studies (table 4). Thus, the most frequently reported endpoint was also the least sufficient endpoint for judging environmental treatment completeness.

This distinction applies across all AOP families. A process can rapidly reduce the measurable concentration of 2,4-D while producing chlorinated phenols, partially oxidized aromatic compounds, short-chain carboxylic acids or other transformation products that persist after the parent herbicide is no longer detected [11–14]. Without carbon-based mineralization data, dechlorination, transformation-product analysis or toxicity testing, such a result supports claims of parent-compound removal or chemical transformation, but not claims of complete treatment or detoxification.

Residual toxicity is therefore not a secondary endpoint for environmental interpretation. It is the evidence domain that determines whether removal and mineralization translate into lower biological hazard. TOC/COD data can support carbon removal or organic-load reduction, but they do not automatically establish reduced toxicity. Conversely, toxicity reduction may occur before complete mineralization if harmful intermediates are transformed or if the bioavailable parent compound is removed. The strongest treatment claims are therefore supported by convergent evidence: selective parent-compound quantification, TOC/COD reduction, transformation-product control and before/after toxicity assessment [33–36]. Because this combined evidence package was uncommon in the corpus, endpoint removal percentages should not be used as a surrogate for treatment completeness.

### 4.3. Mechanistic interpretation and reactive-species evidence

Mechanistic interpretation in AOP studies should not be inferred from removal efficiency alone. A decrease in measurable 2,4-D concentration demonstrates treatment performance, but it does not identify the reactive species, elementary steps or dominant degradation pathway. For this reason, this review distinguishes between observed performance, experimentally supported reactive-species evidence and proposed mechanistic schemes. The main relationships between AOP families, candidate reactive pathways and the type of evidence needed to support mechanistic assignments are summarized in figure 3.

**Figure 3.**
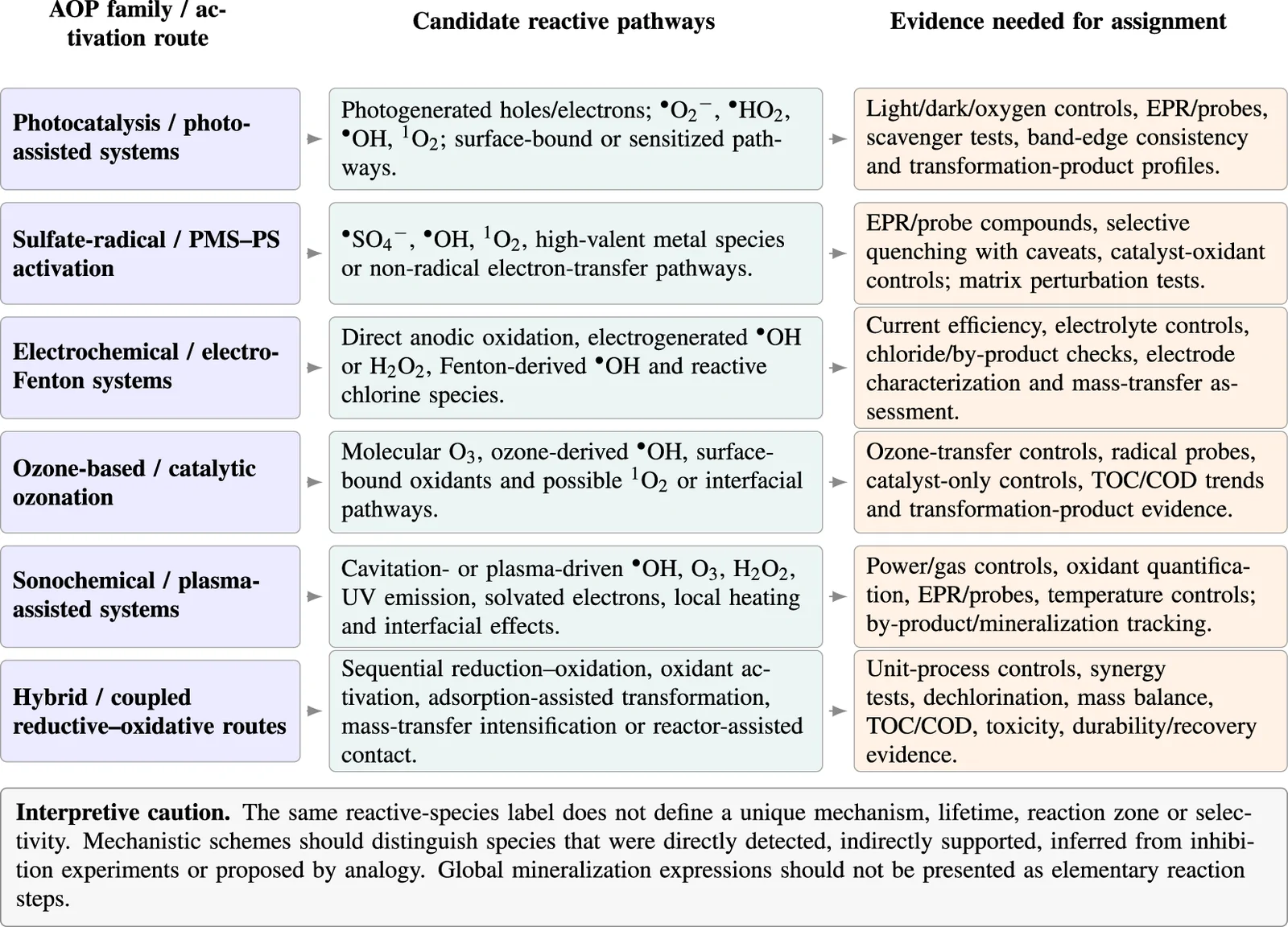
Conceptual map linking AOP families, candidate reactive pathways and the types of evidence needed to support mechanistic assignments in 2,4-D treatment studies. The map summarizes interpretive requirements for reactive-species claims across heterogeneous AOP families and should not be read as a universal reaction scheme.

As shown in figure 3, reactive-species assignment requires evidence beyond removal efficiency alone. The strongest mechanistic assignments are supported by convergent evidence, including electron paramagnetic resonance (EPR) spin trapping, selective scavenging or quenching tests, probe compounds, dechlorination data, transformation-product profiles and kinetic changes under controlled matrix perturbations.

Different AOP families can generate overlapping oxidizing species, but the same species label does not imply the same mechanism, lifetime, reaction zone or selectivity. Hydroxyl-radical systems, sulfate-radical systems, photocatalytic charge-transfer processes, electrochemical oxidation, ozonation, sonochemical cavitation and plasma-assisted treatment involve different combinations of homogeneous, interfacial and surface-mediated reactions. Therefore, ${}^{∙}\mathrm{OH}$, ${}^{∙}{{\mathrm{SO}}_{4}}^{-}$, ${}^{∙}{O_{2}}^{-}$, ${}^{∙}{\mathrm{HO}}_{2}$ and ${}^{1}O_{2}$ should not be treated as interchangeable markers of generic oxidation.

Scavenger tests alone are not definitive, because scavengers may alter pH, ionic strength, adsorption, surface charge or competing reaction pathways. Likewise, transformation products can support a plausible degradation route, but they do not by themselves identify all reactive species responsible for the sequence. This distinction is especially important for sulfate-radical, photocatalytic, electrochemical and hybrid systems. PMS/PS activation may involve ${}^{∙}{{\mathrm{SO}}_{4}}^{-}$, ${}^{∙}\mathrm{OH}$, ${}^{1}O_{2}$, high-valent metal species or non-radical electron-transfer pathways, depending on catalyst composition and water chemistry. Photocatalytic systems may involve photo-generated holes, conduction-band electrons, ${}^{∙}{O_{2}}^{-}$, ${}^{∙}{\mathrm{HO}}_{2}$, ${}^{∙}\mathrm{OH}$, surface-bound species or sensitized pathways. Electrochemical systems may combine direct anodic oxidation, electro-Fenton chemistry, electrogenerated oxidants, reactive chlorine species in chloride-containing media and mass-transfer limitations. Hybrid systems can superimpose several of these routes, making single-species explanations particularly weak unless experimentally supported.

Accordingly, mechanistic schemes should be presented as supported pathways, representative transformations or proposed routes, not as universal elementary sequences. Global mineralization expressions should be treated as overall stoichiometric descriptions. Consistent reactive-species notation reduces ambiguity, but notation is not evidence. Each mechanistic claim should make clear whether a species was directly detected, indirectly supported, inferred from inhibition experiments or proposed by analogy with related systems.

### 4.4. Operational limits: pH, matrix effects and real-water validation

AOP performance for 2,4-D is strongly conditioned by solution chemistry. Model-water experiments are useful for mechanistic control, but they cannot establish practical robustness on their own. pH can affect 2,4-D speciation, catalyst or electrode surface charge, oxidant activation, radical persistence and the stability of Fenton-type or sulfate-radical chemistry. Natural organic matter, alkalinity, inorganic ions, salts, suspended solids and co-contaminants can also change radical lifetimes, light penetration, surface charge, oxidant activation, mass transfer and by-product formation [37,38]. Yet, only 67 of 204 studies reported matrix complexity or real-water validation (table 4), indicating that most of the corpus remains closer to controlled optimization than to deployment-relevant assessment.

Matrix effects are not merely inhibitory; they can suppress, redirect or, in some cases, enhance apparent removal pathways. Natural organic matter and bicarbonate can scavenge highly reactive species and compete for photons or active sites. Chloride can suppress some radical pathways, generate reactive chlorine species or introduce chlorinated by-product concerns, depending on the process. Nitrate, sulfate, carbonate, phosphate and humic substances can modify photocatalytic, electrochemical, ozone-based and sulfate-radical systems in different ways. High removal in ultra-pure or simplified water therefore should not be extrapolated directly to groundwater, surface water, waste water or agricultural drainage [29,39–41].

The family-level comparison shows that matrix validation is unevenly distributed (figure 2; table 5). Sulfate-radical systems reported matrix or real-water validation more frequently than the other larger families, whereas photocatalytic and photo-assisted systems, despite dominating the corpus, reported this domain much less often. This contrast is important because photocatalytic performance depends not only on catalyst composition or band structure but also on turbidity, light screening, fouling, catalyst recovery, natural organic matter and competing ions. Electrochemical and ozone-based systems likewise require matrix-specific interpretation because electrolyte composition, chloride content, alkalinity and conductivity can change both efficiency and by-product profiles.

Future studies should move from simple inhibition tests to staged matrix validation. A defensible sequence would combine model water for mechanistic control, synthetic matrices containing selected ions or scavengers and at least one real-water or well-defined complex matrix. Matrix validation should also be linked to TOC/COD evidence of mineralization or organic-load reduction, transformation products, residual toxicity and energy demand. Without this connection, matrix tests show only that performance changes; they do not show whether treatment remains environmentally meaningful under realistic water conditions.

### 4.5. Energy demand, durability and scale-relevant reactor design

Technology transfer requires more than high parent-compound removal. For AOPs, practical relevance depends on the energy or reagent input required to achieve treatment, the stability of catalysts or electrodes, the risk of leaching or secondary contamination and the extent to which reactor operation can move beyond small-batch tests. In the final corpus, energy or cost metrics were reported in 112 studies, reuse/leaching/durability was coded in 139 studies and reactor scale or transferability was reported in only 37 studies (tables 4 and 6). The reuse/leaching/durability domain should be interpreted cautiously because it often reflected reuse testing alone, not necessarily leaching analysis or post-use characterization. These values show partial progress, but not enough consistency to support a universal technology ranking.

Energy reporting was strongest in electrochemical and plasma-assisted systems, where current density, electrical energy per order, current efficiency or power input are often intrinsic to process description (figure 2; table 5) [13,29–31,42–45]. Even there, direct comparison is limited by differences in system boundaries, treatment volume, initial concentration, target removal level, electrode area, electrolyte composition, gas flow, light source and auxiliary energy assumptions. Photocatalytic studies may report apparent light-driven activity without a complete energy balance; reagent-driven systems may emphasize low electrical demand while omitting oxidant production, dosing, quenching, catalyst synthesis or post-treatment handling. Energy and cost claims should therefore state explicitly which inputs, boundaries and treatment targets are included.

Durability requires the same level of discipline. Reuse tests are informative, but reuse alone is not equivalent to material stability. A catalyst or electrode may retain apparent activity across cycles while releasing metals, changing surface composition, accumulating intermediates, undergoing oxidation-state changes or losing selectivity. Stronger evidence combines reuse performance with leaching analysis, post-use characterization and, where possible, mass-balance or toxicity assessment [38,46–49]. This is particularly important for metal-containing catalysts, heterogeneous Fenton-like systems, doped photocatalysts and electrochemical electrodes.

Reactor design remains one of the weakest links between laboratory performance and application. Most studies still rely on small-batch configurations, whereas transferable operation requires attention to mixing, photon delivery, electrode spacing, mass transfer, oxidant dosing, hydraulic residence time, fouling, separation, catalyst recovery and maintenance. Continuous-flow cells, divided electrochemical systems, membrane-assisted reactors and falling-film configurations are relevant when they improve AOP operation, mass transfer, separation or scale transfer [11,19,32,43,50–52]. The key issue is not the membrane or reactor material in isolation, but whether the configuration provides evidence of treatable volume, residence time, energy demand, durability, matrix robustness and post-treatment safety.

Future reactor-oriented studies should therefore connect degradation performance with TOC/COD evidence of mineralization or organic-load reduction, residual toxicity, energy demand, durability, leaching and realistic matrix testing. Without this combined evidence, scale-relevant claims remain difficult to compare, even when parent 2,4-D removal is high.

### 4.6. Classification of emerging and hybrid systems

Emerging, motion-assisted and hybrid AOP configurations should not be treated as a residual category for processes that do not fit conventional labels. Their classification should depend first on the dominant activation route and then on the function they perform in the treatment sequence: radical or oxidant generation, reductive activation, oxidative transformation, dechlorination, mass-transfer intensification, catalyst recovery, phase separation or reactor-assisted contact between active sites and contaminants [32,50,52–54]. These functions are not equivalent. Grouping systems only because they are recent can obscure whether their contribution is mechanistic, operational, separative, kinetic or reactor-related.

This distinction is especially important for reductive–oxidative and coupled treatment trains. Reductive steps may promote dechlorination or molecular activation, but they do not by themselves demonstrate mineralization or detoxification. A coupled process should therefore be evaluated according to the sequence of treatment functions it supports: parent-compound transformation, dechlorination, oxidation of intermediates, TOC/COD evidence of mineralization or organic-load reduction and residual-toxicity reduction [54–58]. Similarly, motion-assisted, recoverable or reactor-intensified systems should be judged not only by improved contact or apparent removal but also by recoverability, reagent or fuel demand, by-product formation, performance in realistic matrices and scalability.

Plasma-assisted systems illustrate the same classification problem. Plasma treatment can generate multiple oxidants, UV emission, local heating, shock waves and interfacial effects; therefore, its performance cannot be assigned to a single reactive species without supporting evidence [11,31,45,59]. Hybrid systems that combine photocatalysis, ozonation, PS activation, ultrasound, electrochemistry, adsorption or membrane-assisted separation require the same caution [13,32,51,52,60]. Their value depends on whether the combination adds a demonstrable treatment function, such as greater TOC/COD reduction, improved matrix tolerance, lower energy demand, better durability, safer by-product profiles or more transferable reactor operation. Classification by operational function and evidence completeness is therefore more useful than classification by novelty alone.

### 4.7. Research gaps and future directions

The next research need is not merely additional demonstrations of high 2,4-D removal under idealized conditions, but stronger evidence linking removal to mineralization, transformation-product control, residual-toxicity reduction, matrix robustness, energy demand, durability and reactor transferability. A minimum reporting set should include selective parent-compound quantification, TOC or COD evolution, kinetic uncertainty, pH evolution, matrix composition, normalized oxidant dose, catalyst or electrode loading, light or electrical input, transformation-product screening when feasible, residual-toxicity testing before and after treatment and clear treatment-time or residence-time descriptors [11–14,33]. For reusable materials, reuse should be reported together with leaching assessment and post-use characterization.

Mechanistic studies should combine reactive-species evidence with transformation-product and matrix data. Quenching or scavenger tests alone are not sufficient to assign a dominant pathway, especially in systems where ${}^{∙}{{\mathrm{SO}}_{4}}^{-}$, ${}^{∙}\mathrm{OH}$, ${}^{1}O_{2}$, high-valent metals, reactive chlorine species, photo-generated charge carriers or non-radical electron-transfer pathways may coexist. Stronger designs should test whether the proposed mechanism remains consistent in the presence of bicarbonate, chloride, natural organic matter or real-water matrices, and whether it explains mineralization and toxicity changes, not only parent-compound disappearance [37–41].

Technology-oriented studies require standardized energy and reactor reporting. Electrical energy per order, current efficiency, photon flux, reactor volume, treated volume, flow rate, residence time, electrode area, oxidant dose, catalyst loading and system boundaries should be reported in comparable terms. Real-water validation should be treated as a performance requirement, not as an optional final check. Reactor studies should connect degradation efficiency with TOC/COD evidence of mineralization or organic-load reduction, residual toxicity, durability, leaching and matrix effects so that apparent performance can be evaluated against realistic operation [19,29,30,43,44,50].

Data-driven tools, including artificial intelligence, may support future AOP deployment as enabling methods for adaptive control, dose optimization, online monitoring, surrogate modelling and decision support. Their value will depend on the quality of the experimental variables used to train them. Models based only on endpoint removal percentages risk reproducing the same bias identified in this review. Predictive tools will be more useful if datasets include matrix composition, energy input, uncertainty, transformation products, toxicity endpoints, durability and reactor descriptors. They should therefore complement, not replace, mechanistic evidence and complete endpoint reporting.

### 4.8. Limitations of the review

The systematic corpus was restricted to peer-reviewed English-language records captured within the documented 2020–2025 review window and to studies in which 2,4-D was an explicit and extractable target compound in aqueous media. This scope preserved a focused recent synthesis, but it excluded older studies, non-English reports, grey literature and mixed-pollutant studies in which 2,4-D-specific outcomes could not be separated. Three reports sought for retrieval could not be obtained and were recorded as not retrieved. In addition, three included records displayed 2026 issue-year metadata despite being captured during the documented final search workflow; these records were retained as temporal-metadata exceptions and flagged in the supplementary material to preserve traceability.

Google Scholar was included to improve recall, although its interface is less reproducible and less stable over time than Scopus or Web of Science. This limitation was mitigated by documenting the search date, query variants, sorting mode, pages screened and stopping criteria. Nevertheless, Google Scholar results may vary over time. Screening, extraction and scoring were based on information reported in the included articles. Missing TOC/COD evidence, toxicity, leaching, energy or reactor-scale information was not inferred; consequently, the evidence maps reflect both experimental design and reporting completeness.

The evidence-completeness framework was designed for laboratory and bench-scale AOP studies of 2,4-D. It should therefore be interpreted as a structured synthesis tool, not as a universal measure of overall scientific merit. The framework emphasizes domains relevant to environmental treatment completeness and technology transfer, so mechanistically focused studies may receive lower evidence-completeness scores when they do not report several cross-cutting endpoints. Partial scoring also involves judgement when studies use heterogeneous designs and reporting formats. To preserve transparency, the complete study-by-study scoring table is provided in the supplementary material.

A formal meta-analysis was not appropriate because the corpus was highly heterogeneous in initial concentration, matrix composition, reactor geometry, catalyst or electrode material, oxidant dose, irradiation or current regime, treatment time, analytical endpoint and uncertainty reporting. The semi-quantitative synthesis described in §2.8 therefore defines the appropriate level of inference for this review: evidence mapping and cross-family comparison, not pooled-effect estimation or universal technology ranking. These limitations should be considered when interpreting process-family trends and technology-transfer claims.

## 5. Conclusion

This systematic review shows that recent evidence on AOP-based treatment of 2,4-D in aqueous media should be interpreted through treatment completeness, not parent-compound disappearance alone. Across 204 included studies captured within the documented 2020–2025 review window, parent 2,4-D removal was reported almost universally, whereas TOC/COD evidence of mineralization or organic-load reduction, transformation products, residual toxicity, real-water validation, energy demand, leaching, durability and reactor transferability were reported with much lower consistency. A central contribution of this review is, therefore, to move the comparison of AOPs beyond best-case removal percentages towards an evidence-graded assessment of mineralization, detoxification and practical transferability.

Disappearance of 2,4-D should not be used as a surrogate for complete treatment. Mineralization requires carbon-based evidence, and environmental detoxification requires toxicity testing or comparable biological endpoints. Because residual toxicity was reported in only a small fraction of the corpus, claims of environmental safety should be limited to studies that explicitly combine selective parent-compound quantification with TOC/COD reduction, transformation-product control and before/after toxicity assessment.

The process-family comparison did not identify a universally superior AOP route. Photocatalytic and photo-assisted systems dominated the recent literature, but often lacked toxicity, matrix validation and reactor transferability evidence. Sulfate-radical systems more frequently reported selected transformation-product, toxicity and matrix-related descriptors, whereas electrochemical and electro-Fenton systems more consistently reported energy and reactor-relevant metrics. Ozone-based, sonochemical, plasma-assisted and hybrid systems provide useful complementary evidence, but smaller sample sizes limit broad generalization. Process selection should therefore depend on the endpoint being optimized and on the strength of evidence available for that endpoint.

Future studies should adopt a minimum reporting set that includes selective 2,4-D quantification, TOC and, where appropriate, COD evolution, kinetic uncertainty, pH and matrix composition, normalized oxidant or reagent dose, catalyst or electrode loading, light or electrical input, transformation products when possible, residual toxicity, energy metrics, reuse performance, leaching, post-use characterization and reactor descriptors such as treated volume, flow rate and residence time. These variables are essential for making future evidence comparable across AOP families.

Overall, AOPs remain a relevant toolbox for treating 2,4-D-contaminated water, but the field should now shift from demonstrating high removal in simplified systems to producing standardized, mechanism-aware and scale-relevant evidence. The most informative next-generation studies will be those that connect reactive-species generation with mineralization, by-product control, detoxification, real-water performance, energy demand and operational durability.

## Supplementary Material



## Data Availability

This study is a systematic review based on published literature. All data analysed are derived from previously published studies, which are cited in the paper. The complete review workflow and supporting materials, including the search strategy and reproducibility log, PRISMA screening and retrieval audit, full-text assessment, evidence-completeness and reporting-domain scoring, supplementary synthesis tables and included-studies bibliography, are openly available through the Open Science Framework (OSF) [61]. Supplementary material is available online [62].
