# Supplementary material for: Advanced oxidation processes for 2,4-dichlorophenoxyacetic acid in aqueous media: evidence gaps, mechanistic insights and emerging directions
Source: R Soc Open Sci. 2026 Oct 14;13(10):260598. doi: 10.1098/rsos.260598 (PMC13682427; doi:10.1098/rsos.260598)
Supplement: Supplementary file 1 [file rsos.260598.zip › rsos.260598/rsos260598_si_007.docx]

**Electronic supplementary material titles and captions**

*Manuscript RSOS-260598.R1*

Advanced Oxidation Processes for 2,4-Dichlorophenoxyacetic Acid in Aqueous Media: Evidence Gaps, Mechanistic Insights and Emerging Directions

This document provides the descriptive titles and short captions for all six electronic supplementary material (ESM) files submitted with the revised manuscript.

| **File** | **Title** | **Legend/Caption** |
| --- | --- | --- |
| ESM 1 | Search strategy and reproducibility log | Database-specific search strategies, full search strings, search dates, filters, Google Scholar query variants, screening depth and stopping criteria used to define the systematic corpus. |
| ESM 2 | PRISMA screening and retrieval audit | Record-management, Zotero-Rayyan reconciliation, deduplication, PRISMA counts, title/abstract screening decisions, retrieval status and full-text eligibility tracking. |
| ESM 3 | Full-text assessment of retrieved reports | Standardized full-text assessment records for the 217 retrieved reports, including inclusion decisions, exclusion reasons and extracted study-level descriptors. |
| ESM 4 | Evidence-completeness and reporting-domain scoring | Domain-specific evidence-completeness rubric and study-by-study scoring table used to organize reporting completeness and evidence-domain availability for cross-study comparison. This framework is not a formal risk-of-bias assessment or overall study-quality ranking. |
| ESM 5 | Supplementary synthesis tables | Extended family-level and cross-cutting synthesis tables supporting the evidence map, outcome-availability analysis and reporting-gap synthesis. |
| ESM 6 | Included-studies bibliography | Bibliographic record of the 204 studies included in the final systematic review corpus. |

*Note: The files themselves are submitted individually as ESM1-ESM6. This document is provided because the submission form displays caption fields for only five ESM entries.*
